# Invariant community structure of soil bacteria in subtropical coniferous and broadleaved forests

**DOI:** 10.1038/srep19071

**Published:** 2016-01-12

**Authors:** Xiaoli Wang, Xiaoling Wang, Weixin Zhang, Yuanhu Shao, Xiaoming Zou, Tao Liu, Lixia Zhou, Songze Wan, Xingquan Rao, Zhian Li, Shenglei Fu

**Affiliations:** 1 Key Laboratory of Vegetation Restoration and Management of Degraded Ecosystems, South China Botanical Garden, Chinese Academy of Sciences, Guangzhou 510650, China; 2 University of Chinese Academy of Sciences, Beijing 100049, China; 3 Department of Environmental Sciences, University of Puerto Rico, P.O. Box 70377, San Juan, PR 00936-8377, USA

## Abstract

Soil bacteria may be influenced by vegetation and play important roles in global carbon efflux and nutrient cycling under global changes. Coniferous and broadleaved forests are two phyletically distinct vegetation types. Soil microbial communities in these forests have been extensively investigated but few studies have presented comparable data regarding the characteristics of bacterial communities in subtropical forests. We investigated soil bacterial biomass and community composition in three pairs of coniferous and broadleaved forests across a subtropical climatic gradient. We found that bacterial biomass differed between the coniferous and broadleaved forests across the subtropical climate gradient; however, this difference disappeared at some individual sites. In contrast, the same 90 bacterial genera were found in both forest types, and their relative abundances didn’t differ between the forest types, with the exception of one genus that was more abundant in broadleaved forests. Soil nitrogen or moisture was associated with bacterial groups in the coniferous and broadleaved forests, respectively. Thus, we inferred that these forests can respond differently to future changes in nitrogen deposition or precipitation. This study highlights soil bacterial invariant community composition in contrasting subtropical forests and provides a new perspective on the potential response and feedback of forests to global changes.

Forests are one of the major terrestrial ecosystems. Forests play an important role in global carbon sequestration and nutrient cycling, and soil microorganisms are the primary drivers of these ecological processes^1^,^2^. Soils are heterogeneous systems composed of highly diverse microhabitats, and complex patterns in soil microbial communities have been suggested to be driven by plant communities^3^,^4^. Phyletically, forests can be divided into two distinct vegetation types: coniferous and broadleaved forests, each of which shows contrasting characteristics in terms of litter qualities and growth strategies under a changing global environment (e.g., C/N, water and nutrient utilization efficiency)^5^,^6^.

Soil bacteria, the main group of microorganisms found in forests, is enormously diverse, and a single gram of soil may contain one thousand to one million unique “species” of bacteria^7^. Furthermore, soil bacteria are central to the cycling of carbon and nutrients^8^,^9^. The diversity and composition of soil bacterial communities are thought to directly influence a wide range of ecosystem processes^10^,^11^, and bacteria are more sensitive to resource changes, such as nutrients and water, than other soil biota^12^. In recent years, many studies have focused on bacterial diversity and their ecological characteristics for many ecosystems using molecular techniques^13^. Global patterns in bacterial biomass and community structure have been shown to be correlated with aboveground plant productivity, soil organic carbon content, and soil C: N ratios across major global biomes^14^, nevertheless, subtropical forests were typically excluded from analyses due to lack of published data. A quarter of the territory of China (c. 2.5 × 10^6^ km^2^) is in a subtropical climate region, and subtropical forests play an important role in hosting high biodiversity and maintaining ecosystem functioning in China^15^.

The statement “Everything is everywhere, but environment selects” provoked intense discussion in the 21st century investigations of microbial biogeography^16^, and microbial biogeography is controlled primarily by edaphic variables^7^. However, plant type is also considered to be a major factor that affects communities of soil microbes^17^,^18^. The inconsistency of results indicated that there was no clear relationship between soil bacterial communities and vegetation types. The scientific understanding of the relationship between soil bacteria and vegetation type is currently still weak. Phospholipid fatty acids (PLFAs) are now routinely used to estimate soil bacterial biomass, and DNA extracted for molecular analyses has been proposed as another measure for microbial genetic composition^19^. These new techniques allow us now to answer the following questions: (1) Does soil bacterial communities differ between vegetation types? and (2) What are the relative roles of plant vegetation and soil edaphic conditions in controlling bacterial biomass and community structure?

In the present study, we simultaneously compared the soil bacterial biomass and composition during dry and wet seasons in three pairs of coniferous and broadleaved forests and 3 plots per forest type from three sites along the Tropic of Cancer, representing a climatic gradient in subtropical China. Coniferous and broadleaved forests of subtropical China differ in soil organic C content and C: N ratios and these differences are shown to be associated with microbial biomass. Soil organic C has been shown to be correlated with microbial biomass but bacterial composition is not correlated with vegetation across biomes (excluding subtropical forests), with the exception of soil pH^14^. Therefore, we predict that soil bacterial biomass differs, but that composition converges between the coniferous and broadleaved forests along this subtropical climatic gradient.

## Results

### C and N contents in soil and litter

Soil moisture content (SMC), soil total nitrogen (STN) and soil organic carbon (SOC) in the broadleaved forests were generally higher than those in the coniferous forests at all three sites (Table 1). Soil C: N ratio in the coniferous forests was significantly higher than that in the broadleaved forests. Litter mass storage (LS) did not differ between the coniferous and broadleaved forests. However, the litter nitrogen (LN) content in the coniferous forests was significantly lower than that in the broadleaved forests, but the litter organic carbon (LOC) content and litter C: N ratio in the coniferous forests were significantly higher than those in the broadleaved forests at all three sites during both the dry and wet seasons (Table 1).

### Soil bacterial biomass and taxonomic composition

**Figure 1:**
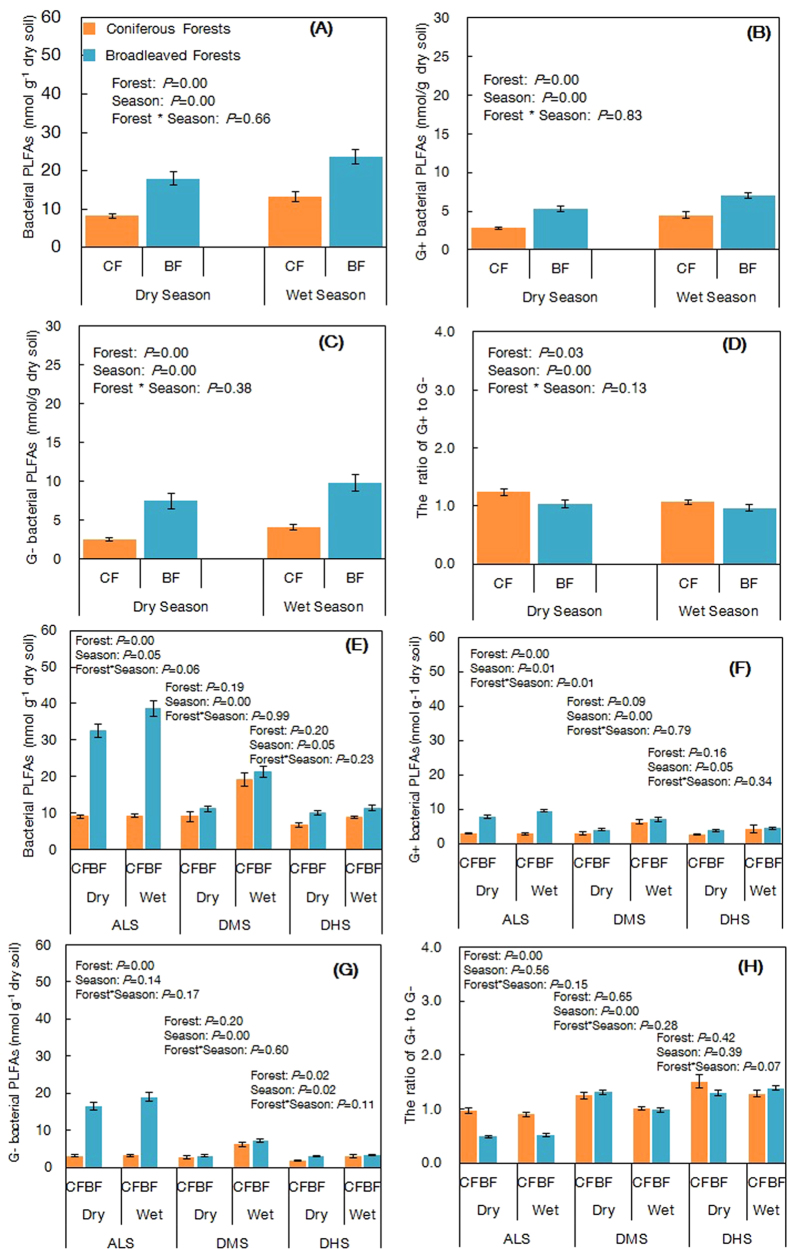
Soil bacterial biomass of the subtropical coniferous and broadleaved forests. (**A**) Total bacterial PLFAs, (**B**) Gram-positive bacterial PLFAs, (**C**) Gram-negative bacterial PLFAs, and (**D**) the ratio of G+ to G− at a 0–15 cm soil depth from all forests in south subtropical China. Values are means + SE, *df* = (1, 17), *n* = 18. (**E**) Total bacterial PLFAs, (**F**) Gram-positive bacterial PLFAs, (**G**) Gram-negative bacterial PLFAs, and (**H**) the ratio of G+ to G− at a 0–15 cm soil depth from two types of forest (coniferous forests and broadleaved forests) at the ALS, DMS and DHS sites. Values are means ± SE, *df* = (1, 5), *n* = 6.

**Figure 2:**
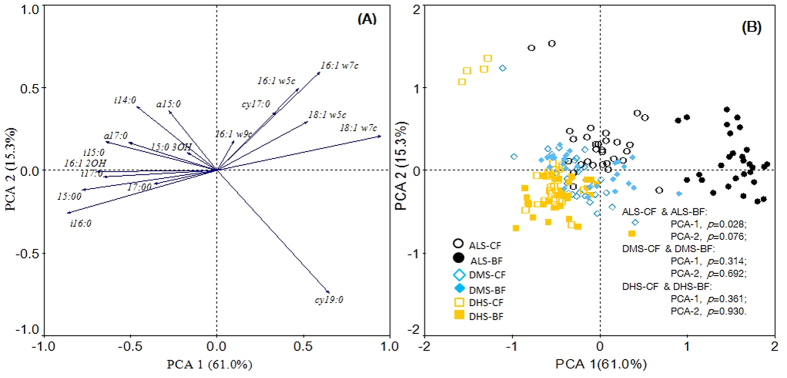
The principal components analysis (PCA) of bacterial PLFA profiles. (**A**) Species distribution of the first two principal components of PLFA profile in the broadleaved forests and coniferous forests, (**B**) Samples distribution of the first two principal components of PLFA profile in the two forests, n = 180. Circles represent the samples of coniferous forests at the ALS site, solid circles represent the samples of broadleaved forests at the ALS site, diamond represents the samples of coniferous forests at the DMS site, solid diamonds represent the samples of broadleaved forests at the DMS site, squares represent the samples of coniferous forests at the DHS site, and solid squares represent the samples of broadleaved forests at the DHS site.

Our PLFA analysis showed that higher total bacterial, gram-negative bacterial, gram-positive bacterial biomass, were found in broadleaved forests and by +117.8%, +198.0% and +86.7%, respectively, compared to coniferous forests in the dry season, and by +79.5%, +135.7%, and 57.7%, respectively, in the wet season (Fig. 1A–C). However, lower ratios of gram-positive (G+) to gram-negative (G−) bacteria were found in broadleaved forests compared to that in the coniferous forests by −16.7% during the dry season, and there was no significantly difference between the two types of forests for the G+ to G− ratio during the wet season along the climatic gradient (Fig. 1D). The repeated measures ANOVA showed that there were significant differences between the two types of forests at the regional scale. At individual sites, total bacteria, G+, and G− bacteria were significantly lower, but the ratio of G+ to G− were significantly higher in the coniferous forests than in the paired broadleaved forests in both seasons at the ALS site. However, there were no significant differences in the three bacterial groups between coniferous and broadleaved forests at DMS and DHS sites, particularly during the wet season (Fig. 1E–G). The ratio of G+ to G− was not significantly different in coniferous forests relative to those in broadleaved forests at the DMS and DHS sites (Fig. 1H). Principal component analysis (PCA) of bacterial PLFAs revealed that PCA_1 and PCA_2 explained 61.0% and 15.3% of variation in bacterial biomass, respectively. The distributions of samples showed converging clusters between these two forests in DMS and DHS sites (Fig. 2).

**Figure 3:**
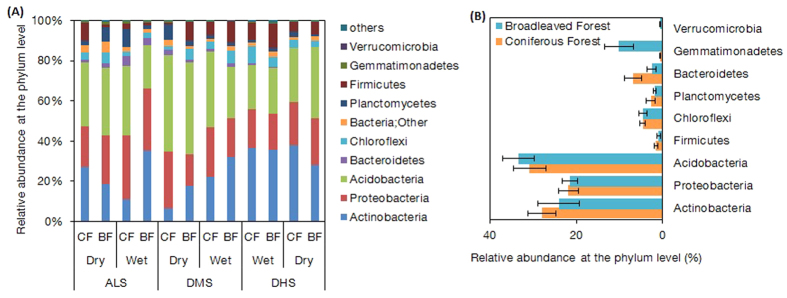
Distribution of the partial sequence of the bacterial 16S rDNA gene at the phylum level from the coniferous and broadleaved forests. (**A**) The bacterial composition of 12 composite samples. The samples were named as Wet-ALS-BF, Wet-ALS-CF, Wet-DMS-BF, Wet-DMS-CF, Wet-DHS-BF, Wet-DHS-CF, Dry-ALS-BF, Dry-ALS-CF, Dry-DMS-BF, Dry-DMS-CF, Dry-DHS-BF, and Dry-DHS-CF, respectively. (**B**) The bacterial composition in the two types of forests, broadleaved forests and coniferous forests. Values are means ± SE (t-test, *df* = 4, *n* = 6). Proportions were calculated based on the pooled sequence classified with an 80% confidence threshold. Phyla accounting for <0.01% of all classified sequences are not shown in the figure.

**Figure 4:**
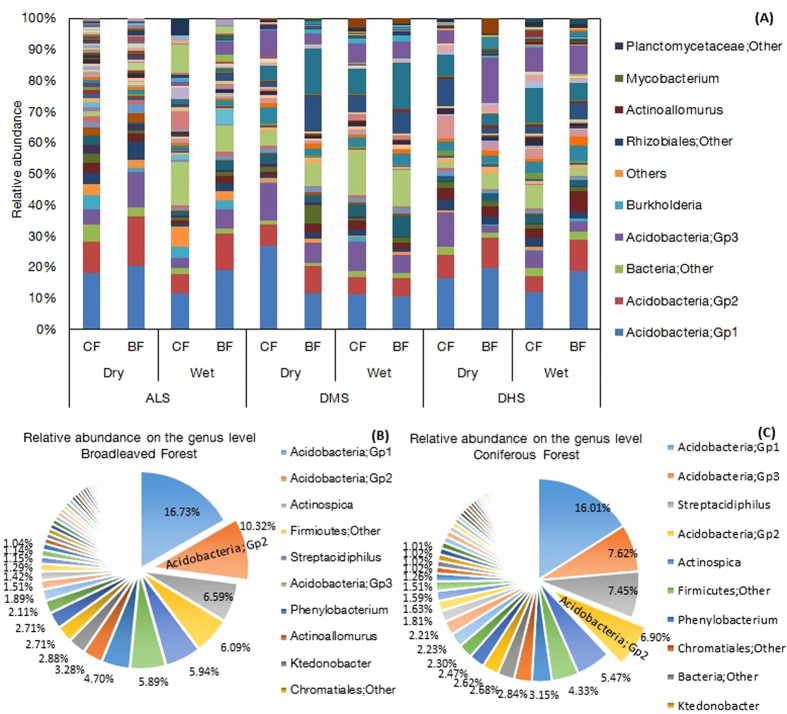
Distribution of partial sequences of the bacterial 16S rDNA gene at the genus level from the coniferous and broadleaved forests. Proportions were calculated based on the pooled sequences classified with 80% confidence threshold. Genera accounting for <0.005% of all classified sequences are not shown in the figure. (**A**) The bacterial composition of 12 composite samples. They were named as Wet-ALS-BF, Wet-ALS-CF, Wet-DMS-BF, Wet-DMS-CF, Wet-DHS-BF, Wet-DHS-CF, Dry-ALS-BF, Dry-ALS-CF, Dry-DMS-BF, Dry-DMS-CF, Dry-DHS-BF, and Dry-DHS-CF, respectively. (**B**) The bacterial composition of broadleaved forests; (**C**) The bacterial composition of coniferous forests. The top ten genera names were listed in the figures.

At the taxonomic level, a total of 89,654 paired-end sequences, ranging from 4,692 to 25,802 reads per composite sample, were obtained from 12 composite samples from these two forests. Random resampling was then performed with 5,690 sequences per sample, resulting in 5,146 OTUs at the 97% similarly level (Supplementary Fig. S1). Next, we obtained a total of 90 identical genera of soil bacteria from 10 phyla across all soil composite samples in the two forests, among which Acidobacteria, Actinobacteria and Proteobacteria accounted for approximately 80% of the bacterial sequences at both the phylum and genus levels (Figs 3A and 4A). We found little difference between the two forests at both the phylum (Fig. 3B; *P* > 0.05) and genus levels (Fig. 4B,C; Supplementary Table S1). Acidobacteria_Gp2 was the only genus whose relative abundant was greater in the broadleaved forests (10.32%) than in the coniferous forests (6.90%) (*P* = 0.043). In contrast, Ktedonobacterales (*P* = 0.016), Solirubrobacterales (*P* = 0.049), Ktedonobacteria (*P* = 0.003) and Thermogemmatispora (*P* = 0.049) were significantly less abundant in the broadleaved forests than in the coniferous forests. However, the relative abundance of these four genera was small and accounted for less than 0.5% in both forests (Supplementary Table S1). Furthermore, the ANOSIM analysis revealed that there was no significant difference in the bacterial community between the broadleaved forests and coniferous forests (*P* = 0.495) at the regional scale, and the result was the same at each site (*P* = 0.333). To further examine differences between these two forests, principal coordinates analysis (PCoA) were performed with the high-throughput sequencing data. Samples of the coniferous forests and broadleaved forests were not well separated from each other (Supplementary Fig. S2).

### Regression between bacterial biomass versus soil and litter properties

**Figure 5:**
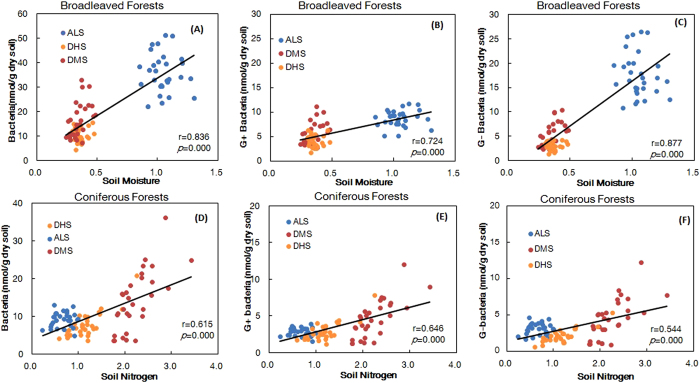
Correlation analysis of soil microbial groups and soil and litter properties. (**A**) Bacteria and soil moisture in the broadleaved forests, (**B**) Gram-positive bacteria and soil moisture in the broadleaved forests, (**C**) Gram-negative bacteria and soil moisture in the broadleaved forests, (**D**) bacteria and soil nitrogen in the coniferous forests, (**E**) Gram-positive bacteria and soil nitrogen in the coniferous forests, (**F**) Gram-negative bacteria and soil nitrogen in the coniferous forests, *n* = 90.

Stepwise regression analysis showed varying associations between bacterial biomass and vegetative (litter) and soil factors for the broadleaved and coniferous forests. In the broadleaved forests at the regional scale, soil moisture content (SMC) was positively correlated with the biomass of total bacteria (coefficient = 27.286) and Gram-negative bacteria (G−) (coefficient = 17.064), but negatively correlated with the ratio of G+ to G− (G+/G−) (coefficient = −0.952). Soil total nitrogen (STN) was positively correlated with Gram-positive bacteria (G+) (coefficient = 0.785). In coniferous forests at the regional scale, STN was not only positively correlated with bacterial biomass (coefficient = 11.014) but also positively correlated with those of G+ (coefficient = 1.620) and G− bacteria (coefficient = 3.117), and soil pH was negatively correlated with G+/G− bacteria ratios (coefficient = −0.413) (Table 2). Our results also showed that the bacterial biomass was positively correlated with SOC (coefficient = 0.156) at the regional scale and that the bacterial biomass was positively correlated with by SOC (coefficient = 0.095) and LTN (coefficient = 3.498) at ALS and DMS sites, respectively, but negatively correlated with pH (coefficient = −11.741) at DHS site (Supplementary Table S2). Linear regression analysis also revealed that there were significant positive correlations between SMC and bacterial biomass, G+ biomass, and G- biomass in broadleaved forests (Fig. 5A–C), and significant positive correlations between STN and bacterial biomass, G+ biomass, and G- biomass in the coniferous forests (Fig. 5D–F).

### Factors that influenced the soil bacterial community composition

**Figure 6:**
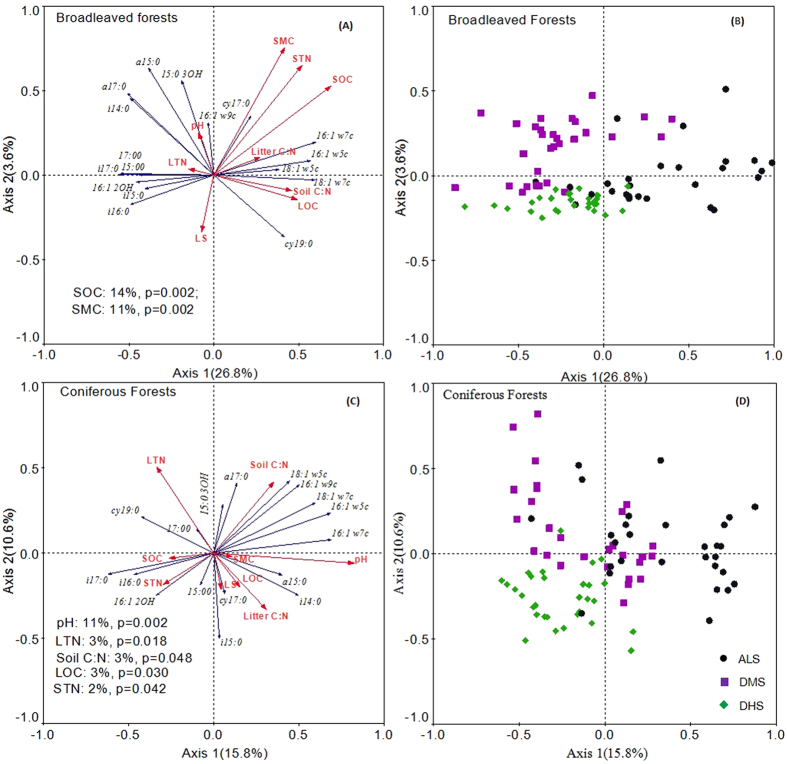
Redundancy analysis (RDA) of PLFA profiles for soil samples using 8 microbial group PLFAs and 9 environmental parameters. Blue line vectors represent microbial variables: bacterial PLFA biomarkers. Red line vectors represent environmental variables: Soil moisture content (SMC), soil organic carbon (SOC), soil total nitrogen (STN), the ratio of soil organic carbon to soil total nitrogen (soil C: N), pH water (pH), litter organic carbon (LOC), litter total nitrogen (LTN), litter storage (LS) and the ratio of litter organic carbon to litter total nitrogen (litter C: N). (**A**) The bacterial communities and the environment factors in the broadleaved forests, *n* = 90; (**B**) distribution of samples in the broadleaved forests, *n* = 90. (**C**) bacterial communities and the environment factors in the coniferous forests, *n* = 90; (**D**) distribution of samples in the coniferous forests, *n* = 90.

RDA analysis indicated that Axis 1 and Axis 2 explained 26.8% and 3.6% of the variation in bacterial PLFAs in the broadleaved forests, and they explained 15.8% and 10.6% in the coniferous forests, respectively. The variations in PLFA profiles in the broadleaved forests were closely correlated with SOC and SMC, which explained 14% and 11% of the variance. In the coniferous forests, the variations in PLFA profiles were closely correlated with soil pH, LTN, soil C: N, LOC, and STN, which explained 11%, 3%, 3%, 3%, and 2% of the variance, respectively (Fig. 6A,C). The distribution of samples in both the coniferous and broadleaved forests showed distinct clusters among the three sites (Fig. 6B,D). SMC, SOC: STN and STN explained the most variance of soil bacterial community at the ALS, DMS and DHS sites, respectively (Supplementary Fig. S3).

## Discussion

Soil microbial communities play critical roles as integral components of forest ecosystem processes^20^. Vegetation type is a major factor in structuring communities of soil organisms among landscapes^3^,^21^. Plant species have profound influences on soil microbial communities, especially when comparing coniferous and broadleaved species^22^,^23^,^24^. However, recent studies indicated that bacterial community composition may be more strongly associated with soil properties, with individual plant species exerting only a weak influence^25^,^26^,^27^.

In the present study, our results showed different biomass of soil bacterial groups between the coniferous and broadleaved forests across the subtropical climate gradient. Apparently, the differences in soil bacterial biomass occurred primarily at an individual site: ALS. The G+/G− ratio, as an indicator of soil starvation stress^28^,^29^, was also higher in coniferous forests than the broadleaved forests at the ALS site. Furthermore, PCA analysis suggested that the soil bacterial community significantly differed between the two types of forests at the ALS site. In contrast, our results showed that there were no differences in biomass for the bacterial groups between the two forests at the DMS and DHS sites. Wan *et al.* (2015) also revealed that bacterial, G+ and G− bacterial biomass in the broadleaved forest (*Mytilaria laosensis*) were almost the same as those in the coniferous forest (*Cuninghamia lanceolata*) based on analysis of PLFA profiles^30^. Additionally, we found that there were no differences for the G+/G– bacteria ratio between the coniferous and broadleaved forests at DMS and DHS sites, which was consistent with another study that was conducted in subtropical forests^31^. Furthermore, a PCA suggested that soil bacterial community did not differ between the two types of forest at both DMS and DHS sites.

To better understand the response of these forests to environmental changes, we examined the relationships between soil bacterial biomass and environmental factors (e.g., nutrients and water availability). If all data were pooled together, SOC, STN, and SMC were found to be the major predictors of soil bacterial biomass, G+ and G− biomass, respectively (Supplementary TableS2). While, soil bacterial biomass was positively correlated with SMC in the broadleaved forests and positively correlated with STN in the coniferous forests across the subtropical gradient. Thus, soil carbon and/or nitrogen effects on soil bacterial biomass may be regulated by SMC at different sites. For example, in the broadleaved forests at the ALS site, soil carbon, nitrogen and water were tightly coupled which may enhance the impact of plant identity on soil microbial community^32^. As a result, the soil bacterial biomass was significant higher than in the coniferous forests. In fact, the results showed that SOC were the major influencing factor on the bacterial biomass at the ALS site (Supplementary Table S2). However, only minor differences in the bacterial biomass were observed between the two forest types at both the DMS and DHS sites. We considered that this contrasting soil bacterial pattern was due primarily to the de-coupling of soil moisture and carbon and/or nutrients that was probably caused by the seasonal soil water deficit^33^. Generally, the hydrothermal conditions in subtropical regions are adequate, but at the ecosystem level soil water availability is seasonally deficient in subtropical forests of China^33^,^34^. Once the soil moisture becomes a limiting factor in an individual site, the effects of plant identities (e.g., carbon and nutrient availability) on soil bacterial biomass may be overridden. As a result, soil bacterial biomass converged in the two types of forests when soil moisture and carbon/nutrients were de-coupled.

Similarly, RDA analysis of PLFA biomarkers showed that soil bacterial community structure is largely invariant between the coniferous and broadleaved forests in subtropical China, but factors correlated with bacterial communities differed between the two forest types. SOC and SMC were correlated with soil bacterial communities in the broadleaved forests, whereas soil pH was correlated with the bacterial communities in the coniferous forests. Forest type effects on soil bacterial community structure may be directly regulated by SMC at different sites. Specifically, the RDA analysis indicated that SMC was the major factor that influenced the soil bacterial community structure at the ALS site (Supplementary Fig. S3). However, the convergence of the soil bacterial community structure was probably due to the limited differences of SMC between the two types of forests at DMS and DHS sites.

In addition, the 16S rRNA analysis demonstrated that the same 90 genera from 10 phyla of bacteria existed in all of the forests with no differences in the relative abundances, with the exception of one genus that was more abundant in broadleaved forests. Specifically, the three major taxa belonging to Acidobacteria, Actinobacteria and Proteobacteria accounted for approximately 80% of the bacterial sequences at the phylum level. Lin *et al.* (2014) also reported that Acidobacteria and Proteobacteria were the most abundant phyla in a hardwood forest and two coniferous forests, with similar relative abundances of various bacterial groups^35^. These results confirmed that bacterial community composition was likely to converge across biomes^14^ and that Acidobacteria predominated in subtropical ecosystems probably because of their adaptation to acidic soil^36^. However, Oh *et al.* (2012) reported that bacterial communities were distinctive in soils under four tropical tree species, with their research focusing on rhizosphere soil^18^. The rhizosphere is widely seen as being chemically and microbiologically distinct from bulk soil^37^,^38^. In one regard, soil samples in the studies conducted by Fierer *et al.* (2009), Liu *et al.* (2012), Lin *et al.* (2014) and us were not separated into rhizosphere soil and bulk soil^14^,^35^,^36^. In contrast, these four tree species from the study of Oh *et al.* (2012) were in a rainforest with the same microclimate and soil types^18^, and this is different from our contrasting, coniferous and broadleaved forests.

Bacteria were fundamentally different from other microbial groups, such as soil fungi, in the characteristics of the physiology and ecology^39^. First, soil bacteria do not have broad symbiotic association with plants with the exception of a few genera such as N_2_-fixing bacteria. In contrast, most plant species have a mycorrhizal association with fungi^40^. Second, bacteria are unicellular organisms, unlike fungi, which can produce the enzymes to degrade the polymers in plant litter. Fungi are generally considered the principal agents in initial degradation of litter, lignin in particular, but most of the soluble products of lignin degradation are probably metabolized by bacteria^41^. Previous study showed that the activity of key enzymes in the degradation of lignin and cellulose (phenol oxidase and cellobiohydrolase) was undetectable in bacteria-only treatment^41^,^42^. Polymeric compounds such as cellulose, hemicellulose, and lignin from litter were not the same, but their decomposing products are chemically less diverse and consist of smaller molecules compounds, polysaccharides and small polymeric chemicals. These few simple and small, chemicals resulted from the middle and late stages of decomposition are then used by bacteria. Thus, plant influences on bacterial community may not be apparent in many ecosystems. Indeed, the ANOSIM analysis demonstrated that the bacterial community (16S rRNA sequences) was highly consistent between the broadleaved forests and coniferous forests.

In conclusion, this study highlighted relatively invariant nature of the bacterial community structure in subtropical coniferous and broadleaved forests. Specifically, bacterial biomass in broadleaved forests were greater than those in coniferous forests as a whole, but this difference disappeared at individual sites due to the de-coupling of moisture and carbon and/or nutrient concentrations. Furthermore, our results showed similar bacterial composition and relative abundances in both types of subtropical forests. Our analyses suggest that changes in nitrogen deposition and precipitation under future global change scenarios may trigger different responses in bacterial communities in the coniferous and broadleaved forests in subtropical China.

## Materials and Methods

### Study sites

The experimental sites were located at the Ailao Mountain Site (ALS), the Daming Mountain Site (DMS), and the Dinghu Mountain Site (DHS), all of which are in the subtropical region of China^37^,^43^,^44^. The ALS, DMS, and DHS are situated near the Tropic of Cancer across the longitude range 100–115 °E in the Yunnan, Guangxi, and Guangdong provinces, respectively (Supplementary Fig. S4). Their annual mean temperature varied from 11.3 °C to 21.4 °C, and annual mean precipitation ranged from 1,103 mm to 2746 mm, representing a climate gradient in subtropical China (Supplementary Table S3). The ground cover of the forests in the subtropical region accounted for almost 100% of the ground surface and included herbaceous plants, ornamental grass, lichen, and litter. The litter layer (3–10 cm thick) almost entirely covered the ground, and the storage of litter in these forests is shown in the Table 1. General characteristics of the forests are shown in the Supplementary Table S3.

### Experimental design and sampling

Three pairs of coniferous and broadleaved forests were chosen from each of the three sites, with three paired forest comparisons and three plots per forest. Soil samples were taken from plots (20 m*20 m) in each forest. There were 18 plots in total and each plot consisted of five subplots (1 m*1 m) for soil sampling. The surface litter of each subplot was removed before taking soil samples and the litter was transported to the laboratory for further analysis. In the dry and wet seasons of 2011, eight soil cores (5 cm in diameter) were randomly taken from each subplot at a depth of 0–15 cm to form a composite sample. The total number of samples for each of the three forest pairs was 180: namely, 3 sites * 2 forest types * 3 replicated plots * 5 subplots * 2 seasons (Supplementary Fig. S5).

### Soil and litter analysis

Soil moisture content (SMC) was measured by oven-drying for 48 h at 105 °C, and soil pH was determined in 1:2.5 (W/V) soil solutions. Soil organic carbon (SOC) and litter organic C (LOC) were determined by dichromate oxidation, and soil total nitrogen (STN) and litter total nitrogen (LTN) were measured with X20A auto digestion and K-06B auto distillation units (Shengsheng Automatic Analytical Instrument co., Ltd, Shanghai, China) based on Kieldahl’s method, a classic technique for measuring the nitrogen content of an organism^45^.

### Phospholipid fatty acid (PLFA) analysis

Phospholipid fatty acids (PLFAs) were analysed according to Bossio and Scow^46^. Concentrations of each PLFA were calculated relative to 19:0 internal standard concentrations. Bacterial biomass was estimated from the concentrations of the 17 PLFAs biomarkers (15:0, 17:0, i14:0, i15:0, i16:0, i17:0, a15:0, a17:0, 16:1ω5c, 16:1ω7c, 16:1ω9c, 18:1ω5c, 18:1ω7c, cy17:0, cy19:0)^46^,^47^, 15:0 3OH and 16:1 2OH^48^, while gram-positive bacterial biomarkers were considered to be represented by the PLFAs i14:0, i15:0, a15:0, i16:0, i17:0, a17:0, and gram-negative bacterial biomarkers included the PLFAs 15:0 3OH, 16:1 2OH, 16:1ω9c, 16:1ω5c, 18:1ω5c, 18:1ω7c, 16:1ω7c, cy17:0, cy19:0^48^. Bacterial biomass was estimated from the concentrations of the 17 bacterial PLFAs and all of them were used to analyse the soil bacterial community.

### DNA extraction, amplification of 16S rRNA genes and pyrosequencing

Genomic DNA was extracted from 0.5 g of the homogenized soil per sample using the PowerSoilTM DNA isolation Kit (MoBio Laboratories, Carlsbad, CA, USA) according to the manufacturer’s instructions. DNA was extracted from all 180 samples, but we mixed these 180 DNA samples into 12 composite samples according to season, site and forest type, namely, 2 seasons * 3 sites * 2 forest types, which were labelled as: Wet-ALS-BF, Wet-ALS-CF, Wet-DMS-BF, Wet-DMS-CF, Wet-DHS-BF, Wet-DHS-CF, Dry-ALS-BF, Dry-ALS-CF, Dry-DMS-BF, Dry-DMS-CF, Dry-DHS-BF, and Dry-DHS-CF. We performed PCR amplification, purification, pooling and pyrosequencing of a region of the 16S rRNA gene^49^. We used the primers F515 (5′-GTGCCAGCMGCCGCGGTAA-3′) and R806 (5′ -GGACTACVSGGGTATCTAAT-3′), which were designed to amplify the V4 hypervariable region and have been demonstrated in *silico* to be universal for nearly all bacterial taxa^50^. A total of 50 ng of template DNA was used for a 50 μl PCR amplification reaction in triplicate following thermal cycling^49^. Replicate PCR reactions for each sample were pooled and purified using a QIAquick Gel Extraction Kit (Qiagen, Chatsworth, CA, USA). A single composite sample for pyrosequencing was prepared by combining approximately equimolar amounts of PCR products from each sample. The amplicons were sequenced using the 454 GS-FLX Titanium protocol (454 Life Sciences/Roche Diagnostics, CT, USA), which yields read lengths of ~400 bp^51^.

### Processing of pyrosequencing data

Raw data were processed and analysed following the QIIME pipelines^52^. A specific barcode was added in the 12 composite samples via the 16S primer to label them during PCR, so that bacterial sequences with the same barcode were assigned to the same composite sample after denoising by denoiserv.0.91^53^. The barcode and primer sequences were removed, and only the first 350 bp after the proximal PCR primer was included for further analysis. Pyrosequences were denoised using the ‘shhh.flows’^54^ (translation of PyroNoise algorithm) and ‘pre.cluster’^55^ commands of the Mothur platform. Chimeric sequences were identified and removed using the UCHIME *de novo* method^56^. Quality sequences were subsequently assigned to samples according to their unique 8-bp barcode and binned into phylotypes using an average clustering algorithm at a 97% similarity level^55^. Representative sequences were aligned using NAST^57^. Taxonomic classification of phylotypes was determined based on the Ribosomal Database Project at the 80% threshold^58^. We estimated the relative abundance (%) of individual taxa within each community by comparing the number of sequences assigned to a specific taxon versus the number of total sequences obtained for that sample. Principal coordinates analysis (PCoA) was used to determine changes of overall bacterial community structure.

### Data analysis

The repeated measures analysis of variance (RM ANOVAs) was employed to determine the effects of two factors (forest and season) and their interaction (forest*season) on soil properties, litter characteristics, and soil bacterial biomass at the regional scale (n = 18; df = (1, 17)) and the local scale(n = 6; df = (1,5)) (Table 1 and Fig. 1). Here, the regional scale represents the subtropical climate gradient in southern China across the longitude range 100–115 °E and the local scale represents the site scale, such as ALS site in our experiment. We performed independent-sample t-tests to compare the differences in bacterial composition at a regional scale (n_1_ = 6, n_2_ = 6, df = 10; Figs 3B and 4B,C and Supplementary Table S1). Here, n_1_ and n_2_ represent the replications of broadleaved forests and coniferous forests, respectively. Stepwise regression and correlation analyses were conducted for the microbial groups, soil properties and litter characteristics (n = 90 at each forest type, Table 2 and Fig. 5; n = 60 at each site, n = 180 at the regional scale, Supplementary Table S3). These statistical analyses were carried out with SPSS 15 (SPSS, Inc, Chicago, IL). To judge the 16S community differences between broadleaved forests and coniferous forests, we conducted ANOSIM analysis using the vegan package within the program R (n_1_ = 6, n_2_ = 6, df = 10). We also did ANOSIM analysis for the 16S community site by site (n_1_ = 2, n_2_ = 2, df = 2). Furthermore, we performed principal component analysis (PCA) of bacterial PFLAs in both forests (n = 180; Fig. 2), and carried out a redundancy analysis (RDA) to determine which environmental factors were related to the composition of the soil bacterial community (n = 90, Fig. 6; n = 60, Supplementary Fig. S3). A statistical significance test for RDA was run using CANOCO software for Windows 4.5 (Ithaca, NY, USA) and tested using Monte Carlo permutation tests (999 permutations). PCoA were performed using the Qiime software (Version 1.7.0). Statistical significance was accepted at *P* < 0.05 level.

## Additional Information

**How to cite this article**: Wang, X. *et al.* Invariant community structure of soil bacteria in subtropical coniferous and broadleaved forests. *Sci. Rep.* **6**, 19071; doi: 10.1038/srep19071 (2016).

## Supplementary Material

Supplementary Information

## Figures and Tables

**Table 1:** Soil and litter properties during the wet and dry seasons at three sites in subtropical China.

Seasons	Sites	Forests	Soil Index					Litter Index			
			SMC (%)	STN(g/kg)	SOC(g/kg)	Soil C: N	pH_water_	LS(kg/m^2^)	LTN(g/kg)	LOC(g/kg)	Litter C: N
Dry	ALS	CF	18.00	0.49	11.01	23.16	5.07	0.53	5.30	491.72	94.64
		BF	105.24	6.80	115.08	17.59	3.67	0.51	10.58	476.86	45.75
	DMS	CF	31.95	2.02	30.22	14.95	4.05	0.62	5.44	522.41	98.81
		BF	29.81	1.74	29.04	16.73	4.01	0.55	13.61	506.93	37.47
	DHS	CF	17.36	0.99	19.91	20.6	3.81	0.45	10.91	493.34	46.96
		BF	38.34	2.10	30.74	14.81	3.64	0.44	18.17	403.98	23.18
Wet	ALS	CF	24.12	0.83	8.54	10.30	5.01	0.46	2.56	422.64	167.37
		BF	104.53	6.05	82.53	13.62	3.94	0.43	6.66	394.42	59.69
	DMS	CF	35.78	2.45	29.67	12.03	4.03	0.53	3.76	443.64	119.42
		BF	38.90	2.88	39.59	13.74	3.92	0.45	7.63	427.12	56.82
	DHS	CF	15.37	1.34	15.38	11.12	3.80	0.47	4.91	432.71	89.59
		BF	32.80	2.47	29.61	11.98	3.63	0.52	8.51	353.83	44.47
Repeated measures ANOVA	Factors		*P*	*P*	*P*	*P*	*P*	*P*	*P*	*P*	*P*
	Forest		**0.00**	**0.00**	**0.00**	0.17	**0.00**	0.17	**0.00**	**0.00**	**0.00**
	Season		**0.04**	**0.00**	**0.01**	**0.03**	0.64	**0.03**	**0.00**	**0.00**	**0.00**
	Forest*Season		0.32	**0.00**	0.54	0.78	0.06	0.78	**0.00**	0.89	**0.00**

Note: Significant effects are shown in bold, as detected by repeated measures ANOVA (*df* = (1, 17), *n* = 18) (*P* < 0.05).

pH_water_, STN, SOC, SMC, LS, LTN, LOC, ALS, DMS, DHS, CF, and BF stand for soil pH, soil total nitrogen, soil organic carbon, soil moisture content, litter storage per square meter, litter total nitrogen and litter organic carbon, Ailaoshan Station, Damingshan Station, Dinghushan Station, coniferous forests, and broadleaved forests, respectively.

**Table 2:** Model summary and ANOVA of the regression in coniferous and broadleaved forests at a regional scale, n = 90.

Forest	Dependent variables	Model	Predictors	R^2^	*F*	*P*
Broadleaved Forest	Bacteria	1	SMC	0.699	204.823	0.000^a^
		2	SMC, litter C: N	0.764	140.470	0.000^b^
		3	SMC, litter C: N, pH	0.780	101.687	0.000^c^
	Gram−	1	SMC	0.770	294.445	0.000^a^
		2	SMC, litter C: N	0.804	178.023	0.000^b^
		3	SMC, litter C: N, pH	0.814	125.418	0.000^c^
	Gram+	1	STN	0.566	114.804	0.000^a^
		2	STN, litter C: N	0.659	84.081	0.000^b^
		3	STN, litter C: N, pH	0.689	63.461	0.000^c^
	Gram+/Gram−	1	SMC	0.753	268.330	0.000^a^
		2	SMC, litter C: N	0.775	150.097	0.000^b^
Coniferous Forest	Bacteria	1	STN	0.269	32.397	0.000^a^
		2	STN, LOC	0.333	21.724	0.000^b^
		3	STN, LOC, pH	0.370	16.841	0.000^c^
		4	STN, LOC, pH, SMC	0.427	15.859	0.000^d^
	Gram−	1	STN	0.265	31.713	0.000^a^
		2	STN, pH	0.393	28.131	0.000^b^
		3	STN, pH, LOC	0.455	23.969	0.000^c^
		4	STN, pH, LOC, SMC	0.484	19.927	0.000^d^
	Gram+	1	STN	0.248	29.023	0.000^a^
		2	STN, LOC	0.294	18.077	0.000^b^
	Gram+/Gram−	1	pH	0.252	29.721	0.000^a^
		2	pH, STN	0.346	23.027	0.000^b^
		3	pH, STN, LOC	0.388	18.144	0.000^c^

Note: Different letters (a,b,c,d) indicate significant differences among different models for the microbial groups (Total bacteria, Gram-positive bacteria, and Gram-negative bacteria in the coniferous forests and broadleaved forests by ANOVA (*P* < 0.05)).

pH_water_, STN, LTN, SOC, LOC, and SMC stand for soil pH, soil total nitrogen, litter total nitrogen, soil organic carbon, litter organic carbon and soil moisture content, respectively.
