# Supplementary material for: Invariant community structure of soil bacteria in subtropical coniferous and broadleaved forests
Source: Sci Rep. 2016 Jan 12;6:19071. doi: 10.1038/srep19071 (PMC4709558; doi:10.1038/srep19071)
Supplement: Supplementary Information [file srep19071-s1.doc]

Title: Invariant community structure of soil bacteria in subtropical coniferous and broadleaved forests

Authors: Xiaoli Wang^1, 2^, Xiaoling Wang^1^, Weixin Zhang^1^, Yuanhu Shao^1^, Xiaoming Zou^1,3^,Tao Liu^1, 2^, Lixia Zhou^1^, Songze Wan^1^, Xingquan Rao^1^, Zhian Li^1^, Shenglei Fu^1,*^

Affiliations:

^1^Key Laboratory of Vegetation Restoration and Management of Degraded Ecosystems, South China Botanical Garden, Chinese Academy of Sciences, Guangzhou 510650, China;

^2^ University of Chinese Academy of Sciences, Beijing 100049, China;

^3^ Department of Environmental Sciences, University of Puerto Rico, P.O. Box 70377, San Juan, PR 00936-8377, USA.

*Corresponding author: Prof. Dr. Shenglei Fu

Key Laboratory of Vegetation Restoration and Management of Degraded Ecosystems,

South China Botanical Garden, Chinese Academy of Sciences,

No. 723, Xingke Road, Tianhe District, Guangzhou 510650, China

E-mail address: [sfu@scbg.ac.cn](mailto:sfu@scbg.ac.cn); Tel.: +86 20 37252722; Fax: +86 20 37252831.

Type of contribution: Original Research;

Date of resubmission: Nov 15, 2015

Number of text pages: 29; Number of words: 6209

Number of tables: 2

Number of figures: 6

Number of supplementary materials: 8

Running title: Invariant bacterial composition in contrasting forests

**Table S1 | the average distribution of partial sequences of bacterial 16S rRNA gene at genus level from the broadleaved (*n_1_*=6) and coniferous forests (*n_2_*=6).** Proportions were calculated based on the pooled sequences classified with 80% confidence threshold. Genus accounting for <0.01% of all classified sequences were not shown in table. Significant effects are shown in bold by independent-sample t-tests (*n_1_*=6; *n_2_*=6, *df*=10; *P*<0.05).

|  | Broadleaved Forests | | Coniferous Forests | | T-test |
| --- | --- | --- | --- | --- | --- |
| No. | Genus name | relative abundance | Genus name | relative abundance | *P* |
| 1 | Acetobacteraceae;Other | 0.53% | Acetobacteraceae;Other | 0.33% | 0.371 |
| 2 | Acidimicrobiales;Other | 0.80% | Acidimicrobiales;Other | 0.97% | 0.530 |
| 3 | Acidisoma | 0.23% | Acidisoma | 0.28% | 0.743 |
| 4 | Aciditerrimonas | 0.28% | Aciditerrimonas | 0.33% | 0.604 |
| 5 | Acidobacteria;Gp1 | 16.67% | Acidobacteria;Gp1 | 15.90% | 0.804 |
| 6 | Acidobacteria;Gp13 | 0.25% | Acidobacteria;Gp13 | 0.12% | 0.136 |
| 7 | Acidobacteria;Gp14 | 0.02% | Acidobacteria;Gp14 | 0.01% | 0.341 |
| 8 | Acidobacteria;Gp2 | 10.28% | Acidobacteria;Gp2 | 6.85% | **0.043** |
| 9 | Acidobacteria;Gp3 | 5.87% | Acidobacteria;Gp3 | 7.57% | 0.404 |
| 10 | Acidobacteria;Gp4 | 0.05% | Acidobacteria;Gp4 | 0.08% | 0.661 |
| 11 | Acidobacteria;Gp6 | 0.17% | Acidobacteria;Gp6 | 0.12% | 0.629 |
| 12 | Acidobacteria;Gp7 | 0.03% | Acidobacteria;Gp7 | 0.12% | 0.425 |
| 13 | Actinoallomurus | 3.27% | Actinoallomurus | 2.45% | 0.370 |
| 14 | Actinobacteria;Other | 0.10% | Actinobacteria;Other | 0.43% | 0.179 |
| 15 | Actinomadura | 0.25% | Actinomadura | 0.17% | 0.399 |
| 16 | Actinomycetales;Other | 0.85% | Actinomycetales;Other | 1.02% | 0.607 |
| 17 | Actinospica | 6.57% | Actinospica | 5.43% | 0.726 |
| 18 | Alicyclobacillus | 1.15% | Alicyclobacillus | 0.03% | 0.057 |
| 19 | Alphaproteobacteria;Other | 0.60% | Alphaproteobacteria;Other | 0.40% | 0.512 |
| 20 | Aminomonas | 0.22% | Aminomonas | 0.05% | 0.094 |
| 21 | Anaeromyxobacter | 0.08% | Anaeromyxobacter | 0.08% | 1.000 |
| 22 | Arthrobacter | 0.83% | Arthrobacter | 0.47% | 0.687 |
| 23 | Bacillus | 0.45% | Bacillus | 0.05% | 0.068 |
| 24 | Bacteria;Other | 1.88% | Bacteria;Other | 2.67% | 0.241 |
| 25 | Betaproteobacteria;Other | 0.20% | Betaproteobacteria;Other | 0.27% | 0.755 |
| 26 | Blastopirellula | 0.10% | Blastopirellula | 0.03% | 0.341 |
| 27 | Bradyrhizobiaceae;Other | 0.28% | Bradyrhizobiaceae;Other | 0.18% | 0.416 |
| 28 | Bradyrhizobium | 0.68% | Bradyrhizobium | 1.02% | 0.129 |
| 29 | Burkholderia | 0.88% | Burkholderia | 1.80% | 0.324 |
| 30 | Burkholderiales_incertae_sedis;Other | 0.07% | Burkholderiales_incertae_sedis;Other | 0.20% | 0.358 |
| 31 | Catenulispora | 0.62% | Catenulispora | 1.58% | 0.342 |
| 32 | Chitinophagaceae;Other | 0.47% | Chitinophagaceae;Other | 0.60% | 0.508 |
| 33 | Chloroflexi;Other | 1.42% | Chloroflexi;Other | 0.97% | 0.220 |
| 34 | Chromatiales;Other | 2.70% | Chromatiales;Other | 2.82% | 0.896 |
| 35 | Chryseobacterium | 0.01% | Chryseobacterium | 0.27% | 0.341 |
| 36 | Clostridia;Other | 0.30% | Clostridia;Other | 0.53% | 0.467 |
| 37 | Conexibacter | 0.48% | Conexibacter | 1.02% | 0.093 |
| 38 | Cystobacteraceae;Other | 0.02% | Cystobacteraceae;Other | 0.23% | 0.259 |
| 39 | Deltaproteobacteria;Other | 0.72% | Deltaproteobacteria;Other | 0.32% | 0.314 |
| 40 | Dyella | 0.52% | Dyella | 0.15% | 0.395 |
| 41 | Ferrimicrobium | 0.17% | Ferrimicrobium | 0.22% | 0.411 |
| 42 | Ferruginibacter | 0.10% | Ferruginibacter | 0.02% | 0.341 |
| 43 | Firmicutes;Other | 6.07% | Firmicutes;Other | 4.30% | 0.479 |
| 44 | Flavitalea | 0.08% | Flavitalea | 0.10% | 0.868 |
| 45 | Gammaproteobacteria;Other | 1.03% | Gammaproteobacteria;Other | 1.00% | 0.950 |
| 46 | Gemmata | 0.85% | Gemmata | 0.72% | 0.810 |
| 47 | Gemmatimonas | 0.52% | Gemmatimonas | 0.57% | 0.799 |
| 48 | Haliangium | 0.05% | Haliangium | 0.10% | 0.568 |
| 49 | Ktedonobacter | 2.87% | Ktedonobacter | 2.60% | 0.785 |
| 50 | Ktedonobacterales;Other | 0.18% | Ktedonobacterales;Other | 0.43% | **0.016** |
| 51 | Ktedonobacteria;Other | 0.07% | Ktedonobacteria;Other | 0.25% | **0.003** |
| 52 | Leifsonia | 0.02% | Leifsonia | 0.10% | 0.341 |
| 53 | Massilia | 0.43% | Massilia | 1.62% | 0.479 |
| 54 | Methylocystaceae;Other | 0.38% | Methylocystaceae;Other | 0.23% | 0.083 |
| 55 | Mucilaginibacter | 0.17% | Mucilaginibacter | 0.33% | 0.365 |
| 56 | Mycobacterium | 2.10% | Mycobacterium | 1.50% | 0.477 |
| 57 | Myxococcales;Other | 0.35% | Myxococcales;Other | 0.43% | 0.381 |
| 58 | Nocardia | 0.68% | Nocardia | 0.70% | 0.967 |
| 59 | Others | 1.28% | Others | 2.22% | 0.429 |
| 60 | Pedomicrobium | 0.13% | Pedomicrobium | 0.05% | 0.399 |
| 61 | Perlucidibaca | 0.08% | Perlucidibaca | 0.02% | 0.217 |
| 62 | Phenylobacterium | 4.68% | Phenylobacterium | 3.13% | 0.477 |
| 63 | Planctomyces | 0.15% | Planctomyces | 0.08% | 0.493 |
| 64 | Planctomycetaceae;Other | 0.72% | Planctomycetaceae;Other | 0.98% | 0.582 |
| 65 | Planctomycetes;Other | 0.20% | Planctomycetes;Other | 0.38% | 0.197 |
| 66 | Proteobacteria;Other | 0.20% | Proteobacteria;Other | 0.30% | 0.418 |
| 67 | Ramlibacter | 0.02% | Ramlibacter | 0.18% | 0.336 |
| 68 | Rhizobiales;Other;Other | 2.70% | Rhizobiales;Other;Other | 2.20% | 0.558 |
| 69 | Rhizomicrobium | 0.68% | Rhizomicrobium | 0.48% | 0.231 |
| 70 | Rhodanobacter | 0.27% | Rhodanobacter | 0.01% | 0.268 |
| 71 | Rhodospirillaceae;Other | 1.50% | Rhodospirillaceae;Other | 1.25% | 0.504 |
| 72 | Rhodospirillales;Other | 0.25% | Rhodospirillales;Other | 0.13% | 0.262 |
| 73 | Rugosimonospora | 0.02% | Rugosimonospora | 0.63% | 0.295 |
| 74 | Selenomonadales;Other | 0.02% | Selenomonadales;Other | 0.15% | 0.046 |
| 75 | Singulisphaera | 0.18% | Singulisphaera | 0.30% | 0.246 |
| 76 | Sinomonas | 0.01% | Sinomonas | 0.35% | 0.286 |
| 77 | Skermanella | 0.07% | Skermanella | 0.08% | 0.721 |
| 78 | Solirubrobacterales;Other | 0.08% | Solirubrobacterales;Other | 0.27% | **0.049** |
| 79 | Stenotrophomonas | 0.01% | Stenotrophomonas | 0.92% | 0.341 |
| 80 | Steroidobacter | 0.37% | Steroidobacter | 0.28% | 0.678 |
| 81 | Streptacidiphilus | 5.92% | Streptacidiphilus | 7.40% | 0.619 |
| 82 | Streptomyces | 0.01% | Streptomyces | 0.03% | 0.145 |
| 83 | Synergistaceae;Other | 0.12% | Synergistaceae;Other | 0.07% | 0.535 |
| 84 | Syntrophaceticus | 0.37% | Syntrophaceticus | 0.62% | 0.329 |
| 85 | Thermoanaerobacteraceae;Other | 0.42% | Thermoanaerobacteraceae;Other | 0.37% | 0.885 |
| 86 | Thermogemmatispora | 0.03% | Thermogemmatispora | 0.17% | **0.049** |
| 87 | Thermoleophilum | 0.02% | Thermoleophilum | 0.13% | 0.272 |
| 88 | Thermomonosporaceae;Other | 0.77% | Thermomonosporaceae;Other | 2.28% | 0.179 |
| 89 | Tumebacillus | 1.13% | Tumebacillus | 0.57% | 0.535 |
| 90 | Zavarzinella | 0.27% | Zavarzinella | 0.18% | 0.658 |

Table S2 | **Model summary and** **ANOVA of the regression for bacterial biomass at the site scale (n=60) and the regional scale (n = 180).** pH_water_, STN, LTN, SOC, LOC, and SMC stand for soil pH, soil total nitrogen, litter total nitrogen, soil organic carbon, litter organic carbon and soil moisture content, respectively.

| Sites | Dependent variables | Model | predictors | R^2^ | *F* | *P* |
| --- | --- | --- | --- | --- | --- | --- |
| ALS | Bacteria | 1 | SOC | 0.477 | 52.824 | 0.000 |
|  |  | 2 | SOC:STN | 0.534 | 32.662 | 0.000 |
|  |  | 3 | LOC | 0.619 | 30.319 | 0.000 |
|  | Gram- | 1 | SMC | 0.787 | 214.684 | 0.000 |
|  | Gram+ | 1 | SMC | 0.836 | 296.372 | 0.000 |
|  |  | 2 | SOC | 0.863 | 178.878 | 0.000 |
|  | Gram+/Gram- | 1 | SMC | 0.702 | 136.900 | 0.000 |
| DMS | Bacteria | 1 | LTN | 0.801 | 233.911 | 0.000 |
|  |  | 2 | LOC: LTN | 0.864 | 180.916 | 0.000 |
|  |  | 3 | SOC | 0.878 | 134.586 | 0.000 |
|  | Gram- | 1 | STN | 0.495 | 56.800 | 0.000 |
|  |  | 2 | LOC | 0.553 | 35.272 | 0.000 |
|  | Gram+ | 1 | STN | 0.521 | 63.035 | 0.000 |
|  |  | 2 | LOC | 0.554 | 35.405 | 0.000 |
|  | Gram+/Gram- | 1 | SOC:STN | 0.245 | 18.809 | 0.000 |
|  |  | 2 | LOC | 0.332 | 14.177 | 0.000 |
| DHS | Bacteria | 1 | pH_water_ | 0.258 | 20.208 | 0.000 |
|  | Gram- | 1 | STN | 0.205 | 14.997 | 0.000 |
|  | Gram+ | 1 | STN | 0.134 | 8.939 | 0.004 |
|  | Gram+/Gram- | n.s. | n.s. | n.s. | n.s. | n.s. |
| Regional Scale | Bacteria | 1 | SOC | 0.215 | 48.692 | 0.000 |
|  |  | 2 | LOC | 0.271 | 32.875 | 0.000 |
|  |  | 3 | LTN | 0.316 | 27.114 | 0.000 |
|  |  | 4 | pH | 0.369 | 25.553 | 0.000 |
|  |  | 5 | SOC:STN | 0.407 | 23.865 | 0.000 |
|  | Gram- | 1 | STN | 0.524 | 195.999 | 0.000 |
|  |  | 2 | SOC | 0.540 | 104.056 | 0.000 |
|  | Gram+ | 1 | SMC | 0.775 | 613.494 | 0.000 |
|  |  | 2 | LTN | 0.796 | 344.646 | 0.000 |
|  | Gram+/Gram- | 1 | SMC | 0.450 | 145.527 | 0.000 |
|  |  | 2 | pH | 0.605 | 135.526 | 0.000 |

**Table S3 | The general characteristics of the three pairs of forest ecosystems in the south subtropical region of China.**

| Sites | Temperature (°C) | Precipitation (mm) | Longitude & Latitude | Altitude (m) | Soil Types | Dominant  Tree species | Dominant understory species |
| --- | --- | --- | --- | --- | --- | --- | --- |
| ALS-BF | 11.3 | 1931 | 24°32'N,102°01'E | 2400–2600 | Typically yellow-brown | *Lithocarpus xylocarpu* | *Sinarundinaria nitida* |
| ALS-CF | 18.7 | 1103 | 24°16'N,100°48'E | 1500-2000 | Mountain yellow red | *Pinus kesiya var. langbianesnis* | *Eupatorium adenophora* |
| DMS-BF | 14.7 | 2746 | 23°24'N,108°31'E | 355-365 | Yellow podzolic | *Quercus griffithii Hook. f. et Thoms ex Miq* | *Indocalamus tessellatus* |
| DMS-CF | 15.1 | 2630 | 23°24'N,108°31'E | 175-365 | Mountain yellow | *Pinus massoniana* | *Dicranopteris dichotoma* |
| DHS-BF | 20.9 | 1927 | 23°10'N,112°31'E | 250-300 | Hydration lateritic | *Cryptocarya concinna Hance* | *Psychotria rubra* |
| DHS-CF | 21.4 | 1927 | 23°10'N,112°31'E | 90-115 | Hydration lateritic | *Pinus massoniana* | *Evodia lepta(Spreng.)Merr.* |

**Figure S1| Rarefaction curves of high-throughput sequencing data of the samples from the broadleaved forests and the coniferous forests.**

**Figure S2| Principal coordinates analysis (PCoA) of bacterial community based on high-throughput sequencing data in the subtropical coniferous and broadleaved forests.** The values for Axes 1 and 2 are percentages of variation attributed to the corresponding axis. Solid circles represent samples of the broadleaved forests, and solid triangle represent samples of the coniferous forests. The blue color represents ALS site, the orange color represents DMS site, and the red color represents DHS site.

**Figure S3| Redundancy analysis (RDA) of PLFA profiles for soil samples using 8 microbial group PLFAs and 9 environmental parameters at the site scale.** Blue line vectors represent microbial variables: bacterial PLFA biomarkers. Red line vectors represent environmental variables: Soil moisture content (SMC), soil organic carbon (SOC), soil total nitrogen (STN), the ratio of soil organic carbon to soil total nitrogen (SOC: STN), pH water (pH), litter organic carbon (LOC), litter total nitrogen (LTN), litter storage (LS) and the ratio of litter organic carbon to litter total nitrogen (LOC: LTN). (A) The bacterial communities and the environment factors at the ALS site, n = 60; (B) The bacterial communities and the environment factors at the DMS site, n = 60; (C) The bacterial communities and the environment factors at the DHS site, n = 60.


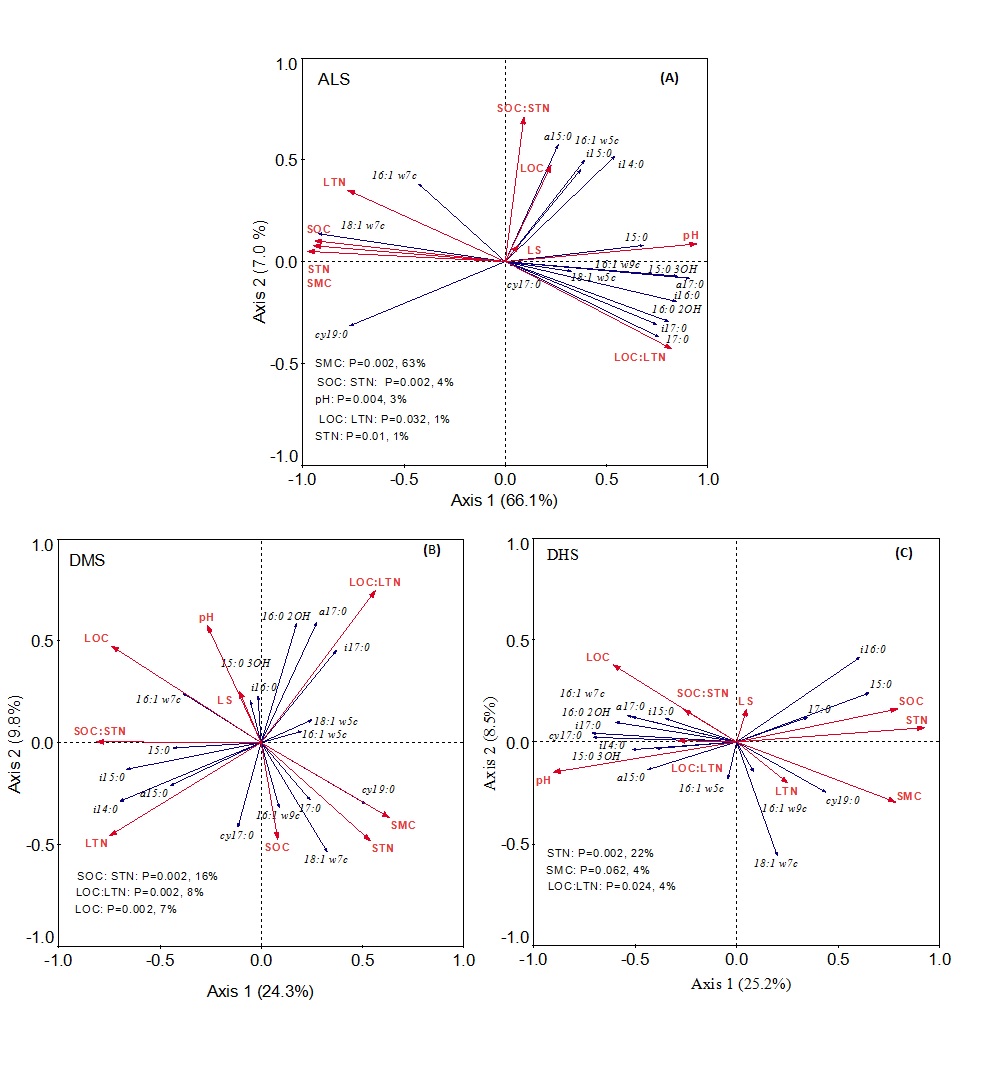


**Figure S4|** Location of nine paired coniferous and broadleaved forests from three study sites in subtropical China. The original map was from Data Sharing Infrastructure of National Administration of Surveying, Mapping and Geoinformation of China. This figure was generated by using Adobe Illustrator CS6 software from Adobe Company (San Jose, California, USA).


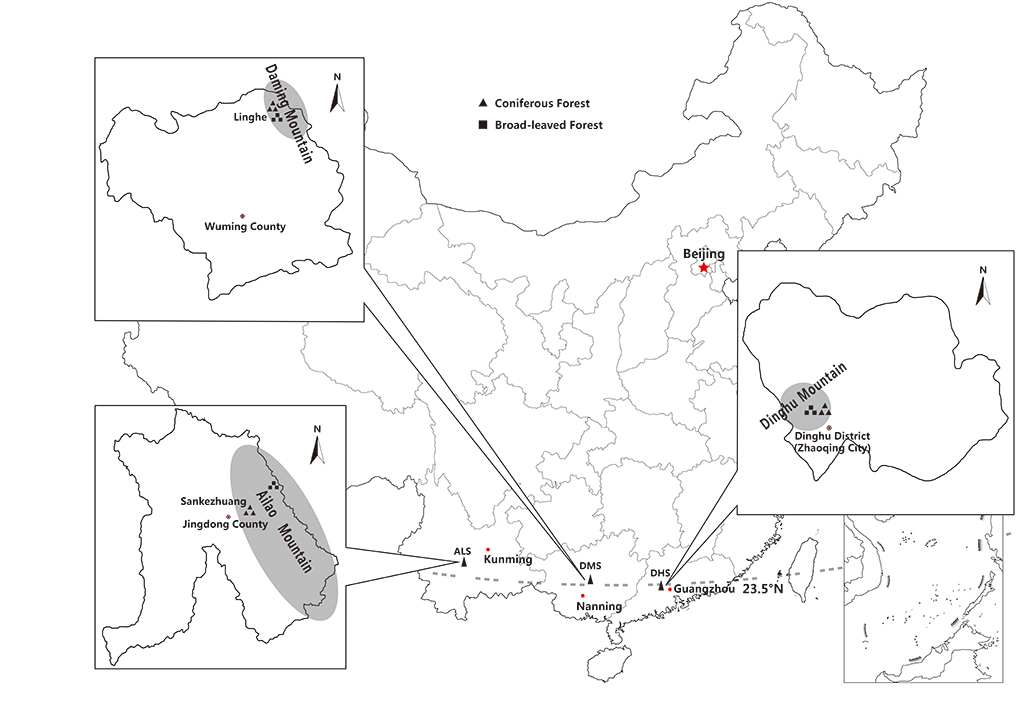


**Figure S5| the diagrammatic sketch of experimental design and sampling.** The total number of samples for each of the nine forest pairs was 180: namely, 3 sites * 2 forest types * 3 replicated plots * 5 subplots * 2 seasons. The 180 samples were used to determinate the bacterial biomass by using PLFAs. We extracted 180 DNA samples and then mixed these 180 DNA samples into 12 composite samples, namely, 2 seasons * 3 sites * 2 forest types. The 12 composite samples were used to determinate the bacterial composition by using 16S pyro-sequencing. The 12 composite samples were named Dry-ALS-CF, Dry-ALS-BF, Dry-DMS-CF, Dry-DMS-BF, Dry-DHS-CF, Dry-DHS-BF, Wet-ALS-CF, Wet-ALS-BF, Wet-DMS-CF, Wet-DMS-BF, Wet-DHS-CF, and Wet-DHS-BF, respectively. ALS, DMS, DHS, Dry, Wet, CF, and BF are the abbreviations of Ailaoshan, Damingshan, Dinghushan, Dry season, Wet season, Coniferous forest, Broadleaved forest, respectively.
